# DNA METHYLATION CLOCKS IN MOUSE

**DOI:** 10.1093/geroni/igz038.3516

**Published:** 2019-11-08

**Authors:** Margarita Meer, Vadim Gladyshev

**Affiliations:** Brigham and Women's Hospital and Harvard Medical School, Boston, Massachusetts, United States

## Abstract

One of important limiting factors in aging research is the time required to measure the effect of an intervention on lifespan. But this situation is now changing due to a recent discovery of DNA methylation-based markers (DNAm clocks). We developed a whole lifespan multi-tissue DNAm clock for mice with R2 =0.89. We also carried out comparative analyses of the available mouse DNAm clocks (single- or multi-tissue, based on different number of sites, based on one genomic locus or multi-loci). In general, tissue specific clocks are more accurate than muti-tissue clocks. We applied these tools to a variety of experimental systems, ranging from interventions to rejuvenation approaches, and analyzed various mouse tissues and public datasets. We further applied DNAm clocks to newly sequenced sets of blood and liver samples. Multi-loci blood clock outperforms other clocks when applied to blood samples, and the liver and multi-tissue clocks show similar precision on liver.

